# Progress in the molecular pathogenesis and nucleic acid therapeutics for Parkinson's disease in the precision medicine era

**DOI:** 10.1002/med.21718

**Published:** 2020-08-06

**Authors:** Dunhui Li, Frank L. Mastaglia, Sue Fletcher, Steve D. Wilton

**Affiliations:** ^1^ Centre for Molecular Medicine and Innovative Therapeutics Murdoch University Murdoch Western Australia Australia; ^2^ Perron Institute for Neurological and Translational Science University of Western Australia Nedlands Western Australia Australia

**Keywords:** blood–brain barrier, molecular pathogenesis, nucleic acid therapeutics, Parkinson's disease, precision medicine

## Abstract

Parkinson's disease (PD) is one of the most common neurodegenerative disorders that manifest various motor and nonmotor symptoms. Although currently available therapies can alleviate some of the symptoms, the disease continues to progress, leading eventually to severe motor and cognitive decline and reduced life expectancy. The past two decades have witnessed rapid progress in our understanding of the molecular and genetic pathogenesis of the disease, paving the way for the development of new therapeutic approaches to arrest or delay the neurodegenerative process. As a result of these advances, biomarker‐driven subtyping is making it possible to stratify PD patients into more homogeneous subgroups that may better respond to potential genetic‐molecular pathway targeted disease‐modifying therapies. Therapeutic nucleic acid oligomers can bind to target gene sequences with very high specificity in a base‐pairing manner and precisely modulate downstream molecular events. Recently, nucleic acid therapeutics have proven effective in the treatment of a number of severe neurological and neuromuscular disorders, drawing increasing attention to the possibility of developing novel molecular therapies for PD. In this review, we update the molecular pathogenesis of PD and discuss progress in the use of antisense oligonucleotides, small interfering RNAs, short hairpin RNAs, aptamers, and microRNA‐based therapeutics to target critical elements in the pathogenesis of PD that could have the potential to modify disease progression. In addition, recent advances in the delivery of nucleic acid compounds across the blood–brain barrier and challenges facing PD clinical trials are also reviewed.

## 1 INTRODUCTION

Parkinson's disease (PD) is the second most prevalent neurodegenerative disease and it is estimated that by 2030 it will affect at least 8 million individuals worldwide.^1^ It was first described by James Parkinson in his landmark 1817 publication: “An Essay on the Shaking Palsy”. In his original description, Parkinson drew attention to the cardinal motor features of the disease: “Involuntary tremulous motion, with lessened muscular power, in parts not in action and even when supported; with a propensity to bend the trunk forward and to pass from a walking to a running pace.”^2^ It has since become apparent that the clinical phenotype is in fact much broader and encompasses a wide range of nonmotor manifestations that includes olfactory and gastrointestinal symptoms, autonomic dysfunction, sleep disorders, depression, and cognitive impairment. Some nonmotor symptoms may manifest in the prodromal stages of the disease and predate the initial motor presentation.^3^, ^4^ Systematic neuropathological studies have shown that the basic pathology and hallmark α‐synuclein containing Lewy body inclusions are not confined to the dopaminergic neurons of the midbrain, but appear to commence in the lower brainstem and olfactory bulb, before extending to more rostral brainstem centers and eventually to the cerebral cortex. It has been proposed that this progression may be due to a prion‐like cell‐to‐cell spread of α‐synuclein pathology.^5^ Since the landmark discovery of the first mutation in the α‐synuclein (*SNCA*) gene in an autosomal dominant PD family,^6^ a variety of mutations in other genes have been identified in autosomal dominant and recessive forms of familial PD that collectively account for around 5%–10% of cases of the disease. In addition, genome‐wide association studies have identified at least 90 common variants associated with risk of PD which are estimated to contribute to between 22% and 27% of the heritable risk of the disease.^7^

Although symptomatic therapies for PD can improve the motor and nonmotor symptoms for patients, these drugs do not stop the ongoing neurodegeneration and progression of the disease, which eventually results in severe motor and cognitive disability and various secondary complications. Thus, there is an urgent need for the development of effective disease‐modifying therapies to slow or arrest the progression of PD. Unfortunately, many promising neuroprotective therapies in experimental animal models of PD have failed to demonstrate efficacy when tested in human clinical trials, and possible reasons for this failure have been discussed elsewhere.^8^ One of the significant challenges is the heterogeneity of clinical phenotypes and genetic‐molecular pathogenesis across the spectrum of sporadic and familial forms of the disease, confounding treatment evaluation in clinical trials. There is thus increasing recognition of the importance of stratifying PD patients into more homogeneous groups according to the genes and/or molecular pathways involved and underlying pathophysiology, to better select patients who may respond to targeted therapies.^9^ More importantly, improved understanding of the molecular pathogenesis is paving the way for novel targeted disease‐modifying approaches, including nucleic acid therapeutics.^10^

Within the last few years, several nucleic acid therapeutic compounds have been approved by the US Food and Drug Administration (FDA) to modify gene expression in a number of inherited conditions including Duchenne muscular dystrophy, spinal muscular atrophy, familial amyloid neuropathy, and Batten disease.^11^, ^12^, ^13^, ^14^ *Milasen*®, an expedited personalized antisense oligomer, was approved by the FDA only 10 months after the genetic cause was identified. This study, by Professor Timothy Yu's group from Boston Children's Hospital, illustrates the efficiency and potential in the nucleic acid therapy discovery process.^14^ The development of different types of nucleic acid therapeutics for PD using experimental in vitro and animal models has attracted increasing interest, with a few compounds under early‐stage clinical trials. Hence, in this review, we will first provide a general update on recent progress in understanding of the molecular pathogenesis of PD and will then focus on the development of precision nucleic acid therapeutics that target the key pathogenetic elements. Overall, it is encouraging that there is great enthusiasm for the development of precision and personalized therapies for PD, particularly for subgroups of patients with distinctive genetic and molecular characteristics, and for whom a more defined target pathway is evident.

## 2 MOLECULAR PATHOGENESIS OF PD

**Figure 1:**
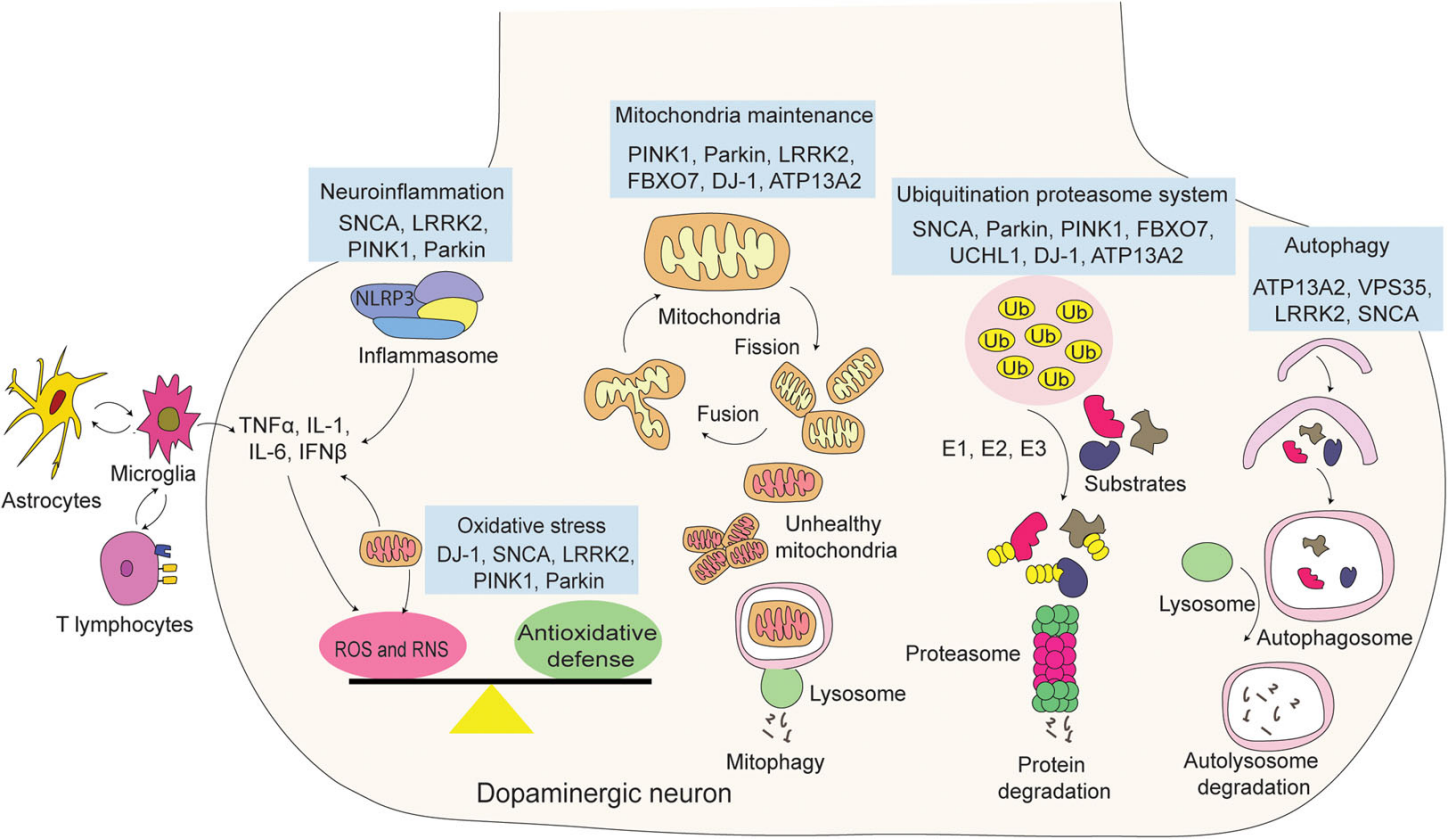
Confirmed causative Parkinson's disease genes and their roles in molecular pathogenesis pathways. E1, E1 ligase; E2, E2 ligase; E3, E3 ligase; NLRP3, nucleotide‐binding oligomerization domain‐like receptor protein 3; ROS, reactive oxygen species; RNS, reactive nitrogen species [Color figure can be viewed at wileyonlinelibrary.com]

The aetiopathogenesis of PD has proven to be complex. The disease was for many years considered to be a sporadic disorder with no genetic associations until the first missense variant in *SNCA* (PARK1 and 4) was identified in 1997.^6^ Since then, a large number of other genetic mutations have been determined to be responsible for familial forms of the disease (Table 1). As a result of these discoveries, several key molecular processes and pathways, including the ubiquitin–proteasomal system, the autophagy–lysosomal pathway, mitochondrial maintenance and integrity, oxidative stress, and neuroinflammation are now known to be involved in PD pathophysiology, as summarized in Figure 1. In addition, other pathways, including innate and adaptive immunity, have also been implicated. In this review, it is not our intent to discuss all of these PD pathways in detail, but rather to provide a general update on progress in some areas of the molecular pathogenesis, of relevance to drug development, and in particular to nucleic acid therapeutics.

**Table 1:** Parkinson's disease related genes and phenotypes

PARK symbol	Gene locus	Gene	Phenotype	Inheritance pattern	Penetrance	Presence of Lewy bodies	Clinical features
Confirmed causative genes							
PARK1 and 4	4q21	*SNCA*	EOPD	AD	40% (duplication); 85% (A53T)	Yes	Rapid progression; high prevalence of dementia and psychiatric complications^15^
PARK2	6q26	*Parkin*	EOPD	AR	100%	No	Slow progression; frequent motor fluctuations; high prevalence of dystonia^16^
PARK6	1p36	*PINK1*	EOPD	AR	100%	One case	Slow progression; good response to levodopa^17^
PARK7	1p36	*DJ1*	EOPD	AR	100%	Unknown	Slow progression; good response to levodopa^15^
PARK8	12q12	*LRRK2*	LOPD	AD	32%–75% (G2019S)	Yes	Slow progression; low prevalence of dementia and psychiatric disturbances^18^, ^19^
PARK9	1p36	*ATP13A2*	EOPD	AR	Incomplete, age‐associated	Unknown	Kufor–Rakeb syndrome; rapid progression^15^
PARK14	22q13.1	*PLA2G6*	EOPD or adult‐onset	AR	Incomplete	Yes	Rapidly progressive parkinsonism with dystonia, and cognitive impairment^20^
PARK15	22q1	*FBXO7*	EOPD	AR	Unknown	Unknown	Variable phenotypes; classical PD ± pyramidal tracts signs^21^
PARK17	16q11.2	*VPS35*	Classical PD	AD	Incomplete, age‐associated	Yes	Parkinsonism; low prevalence of dyskinesia and dystonia^22^, ^23^
PARK19	1p31.3	*DNAJC6*	EOPD	AR	Unknown	Unknown	Slowly progressive; classic PD with mental retardation and seizures^24^, ^25^
PARK20	21q22.2	*SYNJ1*	EOPD	AR	100%	Unknown	Progressive parkinsonism may include seizures, abnormal eye movements, and dystonia^26^
Susceptibility genes that need confirmation							
PARK5	4p13	*UCHL1*	Classical PD	AD	Incomplete	Unknown	Typical idiopathic PD^15^
PARK11	2q37	*GIGYF2*	LOPD	AD	Incomplete, age‐associated	Unknown	Typical idiopathic PD with psychiatric symptoms^27^
PARK13	2p12	*HTRA2*	Classical PD	AD	Low penetrance	Unknown	Typical idiopathic PD; good response to levodopa^28^
PARK18	3q27.1	*EIF4G1*	Classical PD	AD	Incomplete, age‐associated	Yes	Parkinsonism; mild progression; preserved cognitive function ^29^
PARK21	3q22	*TMEM230*	Classical PD	AD	Incomplete (heterozygous mutations)	Yes	Slowly progressive asymmetric parkinsonism^30^
PARK22	7p11.2	*CHCHD2*	Classical PD	AD	Incomplete	Unknown	Typical idiopathic PD; good response to levodopa^31^
Risk factor gene							
NA	1q21	*GBA*	NA	AD/AR	Incomplete, age‐associated	Yes	Accelerated progression and high risk of cognitive impairment^32^, ^33^

Abbreviations: AD, autosomal dominant; AR, autosomal recessive; EOPD, early‐onset PD; LOPD, late‐onset PD; NA, not applicable; PD, Parkinson's disease.

### 2.1 Ubiquitin–proteasome system

The discovery that Lewy bodies are ubiquitin‐immunopositive provided an early indication that the ubiquitin–proteasome system (UPS) plays a role in PD pathogenesis.^34^ As the main component of Lewy bodies, monomeric or α‐helically folded tetrameric α‐synuclein is actively degraded by the UPS under physiological conditions.^35^, ^36^ *SNCA* mutations, including duplication or triplication of the whole gene, or disease‐associated missense mutations, render α‐synuclein prone to misfolding and formation of toxic protein aggregates. These aggregates have prominent inhibitory effects on 20S/26S proteasomal protein cleavage in dopaminergic cells,^37^ which in return further accumulates aggregates of toxic α‐synuclein.^38^

Parkin is an auto‐inhibited RING‐between‐RING E3 ligase in the UPS which is deficient in autosomal recessive PD (PARK2). Upon activation by PINK1, Parkin undergoes a conformational change that facilitates its ubiquitin ligase activity.^39^ Amongst the many Parkin substrates, aminoacyl‐tRNA synthetase complex interacting multifunctional protein‐2 accumulates when Parkin is deficient, which activates poly(ADP‐ribose) polymerase‐1 (PARP1) and causes selective loss of dopaminergic neurons.^40^ Consequently, PARP1 inhibitors, which have been approved by the FDA for certain breast and ovarian cancers, are now being considered as repurposed drugs for the treatment of PD.^41^ PINK1 has multiple functions, including modulating mitochondrial respiratory chain activity, regulating neuroinflammation, and promoting neuron survival.^42^ In terms of its roles in the UPS, cytosolic PINK1 phosphorylates some Parkin substrates, primes Parkin‐mediated ubiquitination, and ultimately facilitates the degradation of pathological proteins.^43^

The efficient and timely ubiquitination for substrate degradation requires the maintenance of cellular ubiquitin homeostasis. As a PD susceptibility gene,^44^ the ubiquitin C‐terminal hydrolase L1 (UCHL1, PARK5) is one of the most abundant deubiquitinating enzymes that is predominantly expressed in the brain. UCHL1 is highly efficient in cleaving monoubiquitin from small peptides that are conjugated to the C‐terminus of a ubiquitinated protein.^45^, ^46^ UCHL1 also participates in other pathways including processing of proubiquitin, E3 ligase function, maintaining axonal function, and inhibiting autophagy.^47^, ^48^, ^49^, ^50^ Considering its multiple roles in the UPS and other cellular functions, UCHL1 might be considered as an attractive therapeutic target for PD and related disorders.

### 2.2 Autophagy–lysosomal pathway

Autophagy is a catabolic process that delivers dysfunctional organelles or misfolded proteins to the lysosome for degradation. With substantial evidence that proteins encoded by PD causative or risk genes directly or indirectly regulate the autophagy–lysosomal pathway, dysregulated autophagy is believed to play a major role in PD pathogenesis. Emerging studies are showing that multiple variants in lysosomal storage disorder genes can contribute to PD susceptibility.^51^, ^52^ Deficiency of the lysosomal hydrolase glucocerebrosidase, encoded by *GBA*, leads to the most common lysosomal storage disorder, Gaucher disease. Generally, around 5%–15% of PD patients are reported to carry heterozygous *GBA* mutations. However, the prevalence of *GBA* mutations varies in different populations, with the highest prevalence being in Ashkenazi Jewish PD patients (20%).^53^ While, it is clear that *GBA* mutations cause autophagy–lysosomal dysfunction, the specific mechanisms involved remain unclear.^54^

Mutations in ATP13A2, a transmembrane endo/lysosomal P‐type transport ATPase, cause Kufor–Rakeb syndrome (PARK9), a rare subtype of juvenile‐onset autosomal recessive parkinsonism. ATP13A2 controls lysosome homeostasis and regulates autophagosome–lysosome fusion through HDAC6 that is recruited to the lysosome and facilitate autophagy flux.^55^ Moreover, ATP13A2 interacts with endocytic signaling lipids through its hydrophobic N‐terminal to enable endocytic cargo export.^56^ Another PD‐related gene product that regulates endosomal–lysosomal trafficking is vacuolar protein sorting protein‐associated protein 35 (VPS35, PARK17), a member of the retromer complex. This complex is involved in intracellular trafficking of proteins including α‐synuclein and proteins that are important for autophagosome formation.^57^ Reduced α‐synuclein degradation and profuse Lewy body pathology in the substantia nigra and other midbrain region were found in patients with *VPS35* mutations.^58^

The major pathogenic factor in both familial and sporadic PD is considered to be the formation of α‐synuclein aggregates, which is due in part to malfunction of the α‐synuclein degradation machinery. Normal monomeric α‐synuclein is usually degraded by chaperone‐mediated autophagy, while macroautophagy has been implicated in the clearance of α‐synuclein oligomers.^59^ The burden of toxic α‐synuclein aggregates, may compromise the macroautophagy pathway through interfering with autophagosome formation or clearance.^60^ On the other hand, when macroautophagy is affected or inhibited by gene mutations, intracellular α‐synuclein clearance is impeded and accumulation of the mutant protein is exacerbated. Mutations in *LRRK2*, which are the most common cause of familial PD (PARK8), have also been reported to dysregulate macroautophagy, although the findings in different studies have been somewhat contradictory.^61^

### 2.3 Mitochondrial maintenance

The landmark observation that parkinsonism could result from the accidental intake of 1‐methyl‐4‐phenyl‐1,2,3,6‐tetrahydropyridine, a potent inhibitor of mitochondrial complex I, was a critical finding that first implicated mitochondria in the pathogenesis of PD.^62^ Since then, many aspects of mitochondrial dysfunction have been investigated, including impaired mitochondrial maintenance, defective mitophagy, calcium imbalance, oxidative stress, and effects of neuroinflammation.^63^ LRRK2 is thought to act as a scaffold during mitochondrial fusion and fission, with the WD40 repeat‐containing domain and the dynamin‐related GTPase domain required for LRRK2 interacting with mitochondrial fission or fusion factors.^64^, ^65^ While, PINK1 and Parkin contribute to mitochondrial fission and fusion through ubiquitinating mitochondrial fusion regulators, including mitofusin 1/2.^66^

Mitophagy is a cargo‐specific form of autophagy that removes damaged or excessive mitochondria to maintain an optimally functioning mitochondrial network. PINK1 is ubiquitously expressed in all mitochondria and maintained at a low level in healthy mitochondria.^67^ Upon mitochondria damage or depolarization, PINK1 proteolysis is impeded. Subsequently, the overexpressed PINK1 recruits Parkin from the cytosol into mitochondria,^68^, ^69^ then mediates Lys63‐linked ubiquitination of outer mitochondrial membrane proteins and induces mitophagy. Loss‐of‐function mutations in the PINK1/Parkin pathway result in impaired mitophagy. The accumulation of dysfunctional mitochondria cause increased reactive oxygen species (ROS) generation, cytochrome c leakage, and mitochondrial DNA mutations, ultimately leading to dopaminergic neuron death.^70^

Several other PD pathogenic genes are also reported to exert effects on the mitophagy pathway. A recent study found that *LRRK2* mutations interfere with the interaction between dynamin‐related protein 1 and its mitochondrial receptor MiD51, which is required for the initiation of Parkin‐dependent mitophagy.^71^ Under basal conditions, the outer mitochondrial membrane protein Miro is rapidly removed by LRRK2 to stop mitochondrial mobility and facilitate mitophagy,^72^ while mutated LRRK2 disrupts this function, delays the arrest of damaged mitochondria and consequently hinders the induction of mitophagy.^73^ Other factors such as the accumulation of α‐synuclein, ATP13A2 deficiency, and GBA mutations have also been shown to impair mitophagy.^74^, ^75^, ^76^

In addition to their bioenergetic functions, mitochondria fine‐tune cytosolic Ca^2+^ levels by controlling Ca^2+^ uptake and efflux through several calcium transporters.^77^, ^78^ Mutations in *SNCA*, *PINK1*, and *LRRK2* have been shown to dysregulate Ca^2+^ transporters, resulting in mitochondrial Ca^2+^ overload, increased ROS production, and ultimately neuronal cell death.^77^, ^79^, ^80^, ^81^ The tethering complexes connecting mitochondria and the endoplasmic reticulum are crucial for cellular calcium handling,^82^ and mutations in *SNCA*, *PARKIN*, and *DJ1* have been found to interfere with the coupling of the complexes and then impair Ca^2+^ dynamics.^82^, ^83^, ^84^, ^85^ Besides the role of PINK1, Parkin and DJ1 in mitophagy and Ca^2+^ dynamics, these proteins also modulate the function of the mitochondrial respiratory chain complex.^86^ Mutations in these genes disrupt the assembly of the components of the respiratory chain complex, inhibit complexes I and III, and increase the generation of ROS and reactive nitrogen species (RNS).

### 2.4 Oxidative stress

Oxidative stress is a cascade reaction resulting from an imbalance between increasing free radicals, including ROS and RNS, and cellular antioxidant activity. The accumulation of free radicals causes DNA and protein oxidation, or lipid peroxidation that compromises cell integrity and leads to cell death. As neurons have a high demand for oxygen but relatively low levels of antioxidants, they are particularly susceptible to oxidative stress.^87^, ^88^ The impairment in mitochondrial homeostasis in PD could exacerbate the production of ROS that are the by‐products from the oxidative phosphorylation pathway. Some PD‐related gene products contribute to mitochondria‐related oxidative stress through regulating mitochondrial homeostasis or mitophagy as discussed above. In addition, α‐synuclein binds directly to mitochondria, impairing mitochondrial protein import and resulting in deficient mitochondrial respiration and enhanced ROS production.^89^ Nonfibrillar phosphorylated α‐synuclein is a newly identified α‐synuclein neurotoxic isoform, and its aggregates have been shown to induce mitochondrial fragmentation via reduced lipoylation and aggravate oxidative stress.^90^

The protein deglycase DJ1, encoded by PARK7, is ubiquitously expressed in the brain and acts as a sensor of oxidative stress.^91^ DJ1 is involved in several signaling pathways that are protective against oxidative injury, including activating the extracellular signal‐regulated protein kinases 1 and 2 and Akt pathways,^92^, ^93^, ^94^ inhibiting apoptosis signal‐regulating kinase 1,^95^ and regulating transcription factors such as p53, nuclear factor kappa B, and nuclear factor erythroid 2–related factor 2.^91^, ^96^, ^97^, ^98^ The crosstalk between DJ1 and other major antioxidant systems, such as the thioredoxin system and the glutathione system^91^, ^99^ further supports the cellular antioxidant activity to provide an optimal response to oxidative stress. DJ1 mutants could produce selective mitochondrial pathologies that impair Ca2^+^ dynamics and free radical homeostasis through disrupting the interaction between DJ1 and mitochondrial accessory proteins.^84^

### 2.5 Neuroinflammation

Since the first report of reactive microglia in postmortem PD brain tissue in 1988,^100^ microglial activation and high levels of proinflammatory cytokines have been consistently detected in PD brains.^101^ There is, therefore, convincing evidence that neuroinflammation is involved in dopaminergic neuron death and neurodegeneration.^102^ As the primary central nervous system (CNS) resident immune cells,^103^ microglia can be activated to yield two different phenotypes (M1/M2) to produce either proinflammatory or anti‐inflammatory cytokines to maintain CNS homeostasis. Chronic activation by misfolded proteins or environmental toxins results in M1 microglia that produce proinflammatory cytokines, including tumor necrosis factor‐α (TNF‐α), interleukin‐1β (IL‐1β), and interferon‐γ. The initial microglial activation and subsequent production of proinflammatory mediators may also result in activation of astrocytes that are considered to be integrative regulators of neuroinflammation in the nervous system.^104^

Apart from the role of innate immunity in neuroinflammation, there is increasing evidence that adaptive immunity also plays a role in PD pathogenesis.^105^, ^106^ An increase in the number of cytotoxic and memory T cells, and changes in proportions of helper T cells and naive T lymphocytes in peripheral blood, indicates the activation of a cytotoxic immune response in PD.^106^, ^107^, ^108^ Infiltration of T cells in the substantia nigra of PD mice was reported to affect neurodegeneration in other ways, including inducing microglial activation to the M1 phenotype.^109^, ^110^ Although the mechanism of T cell invasion into the CNS is still uncertain, microglia as the brain‐resident macrophages are believed to present CNS‐derived antigens to peripheral T lymphocytes, resulting in T cell activation and infiltration.^111^, ^112^

Along with the cellular mediators, several molecular mechanisms are involved in modulating neuroinflammation. Extracellular wild‐type or pathological α‐synuclein activates microglia through toll‐like receptor 2 and the Janus kinase–signal transducers and activators of transcription signaling pathways, which produces various proinflammatory cytokines and chemokines.^113^, ^114^, ^115^ Blocking the toll‐like receptor 2 signaling alleviates α‐synuclein accumulation, neuroinflammation, and behavioral deficits in the α‐synuclein transgenic mouse model.^116^ LRRK2 interacts with and phosphorylates nucleotide‐binding oligomerization domain‐like receptor C4, an inflammasome protein, and subsequently activates caspase‐1.^117^ In addition, the LRRK2 G2019S mutation appears to drive microglia towards the reactive phenotype with enhanced inflammatory responses^118^ and increases PD susceptibility.^119^

PINK1‐ and Parkin‐mediated mitophagy can prevent mitochondrial‐induced inflammation.^120^ Deficiency in either PINK1 or Parkin function results in elevated levels of cytosolic and circulating mitochondrial DNA, or double‐stranded mitochondrial RNA that promotes proinflammatory responses through different mechanisms.^121^, ^122^ Apart from roles in mitochondrial‐induced inflammation, PINK1 and Parkin also contribute to neuroinflammation via separate pathways. Through promoting proteasomal degradation of TNF‐α receptor‐associated factor 2/6^123^ or regulating ubiquitin‐modifying enzyme A20 and NLRP3‐inflammasome, Parkin suppresses inflammation and cytokine‐induced neuron death.^124^, ^125^ PINK1 modulates neuroinflammation in a glial cell type‐specific manner, with studies showing that loss of PINK1 increases proinflammatory cytokines (TNF‐α, IL‐1β, and NO) in astrocytes.^126^

The progress in the molecular findings is gradually leading to the clarification of PD pathogenesis and is providing justifications to develop molecular pathway targeted therapeutics. Although most of the pathways are closely related and/or overlapping, some pathways are proposed to result in a distinct disease process and resultant phenotypes. For example, mitochondrial damage is a particular problem in the substantia nigra,^127^ and thus for patients with mitophagy failure, their clinical features are hypothesized to have a more dopaminergic nature. However, for those who have a dysregulated autophagy–lysosomal pathway, the clinical features might be more generalized.^128^, ^129^ The genes and molecular events involved are somewhat clearer in monogenic familial PD cases, which is facilitating the development of precision or personalized treatment strategies. Nucleic acid therapies are particularly attractive in this scenario since approaches including small interfering RNAs (siRNA), microRNAs (miRNA) and antisense oligonucleotides (ASO) can be applied to tackle some gain‐of‐function mutations, while splice‐switching antisense oligomers can be used to address selected loss‐of‐function mutations. Some common mutations of PD genes and potential nucleic acids‐based therapeutic strategies are summarized in Table 2.

**Table 2:** Current status of nucleic acid therapeutic strategies for the common genetic forms of PD

Genes	Common mutations	Mechanism	Potential therapeutic strategies	Current Status
SNCA	c.88G>A (p. A30P), c.152G>A (p. E46K), c.157G>A (p. A53T), increased copy number	Gain‐of‐function	Downregulation (ASO, siRNA, shRNA); Splice switching (ASO)	Under development (in vitro and in vivo)
Parkin	exon 3 deletion, exon 4 deletion, c.838G>A (p. A280A)	Loss‐of‐function	Exon skipping (ASO)	Under development (in vitro)
PINK1	c.1231G>A (p. G411S), c.1311G>A (p. T437X)	Loss‐of‐function	Upregulation (ASO)	N/R
DJ1	c.293G>A (p. A98G), c.310G>A (p. A104T)	Loss‐of‐function	Upregulation (ASO)	N/R
LRRK2	c.6055G>A (p. G2019S), c.4322G>A (p. A1441H)	Gain‐of‐function	Knockdown (ASO, siRNA, miRNA)	Phase I clinical trial
ATP13A2	No recurrent mutations	Loss‐of‐function	Exon skipping (ASO); Upregulation (ASO)	N/R
GBA	c.1226A>G (p. N370S), c.1448T>C (p. L444P)	Gain‐of‐function^a^	Downregulation (ASO, siRNA, miRNA, shRNA)	Under development

Abbreviations: ASO, antisense oligonucleotides; miRNA, microRNA; N/R, no reports; PD, Parkinson's disease; shRNA, short hairpin RNA; siRNA, small interfering RNA.

^a^ Both gain‐of‐function and loss‐of‐function mechanisms are proposed for GBA‐associated Parkinsonism.

## 3 NUCLEIC ACID THERAPEUTICS AND ITS PROGRESS IN PD RESEARCH

Since the FDA first approved the nucleic acid drug, Vitravene in 1998 to treat cytomegalovirus retinitis in immunocompromised patients, nucleic acid therapeutics have gradually come‐of‐age. In excess of 200 nucleic acid drug clinical trials are registered at ClinicalTrials.gov and over 10, 000 patients have received nucleic acid drugs since 2016.^130^ Nucleic acid therapeutics are emerging as a well‐established, validated class of drugs that could manipulate almost any gene with high specificity.^131^ However, there are a number of hurdles that need to be overcome before successful widespread clinical application of nucleic acid compounds. The first and foremost is the instability of unmodified natural nucleic acids. As expected in normal RNA and DNA metabolism, the natural phosphodiester backbone is particularly sensitive to degradation by endonuclease and exonucleases.^132^, ^133^, ^134^

### 3.1 Nucleic acid chemistries, modifications, and related toxicities

**Figure 2:**
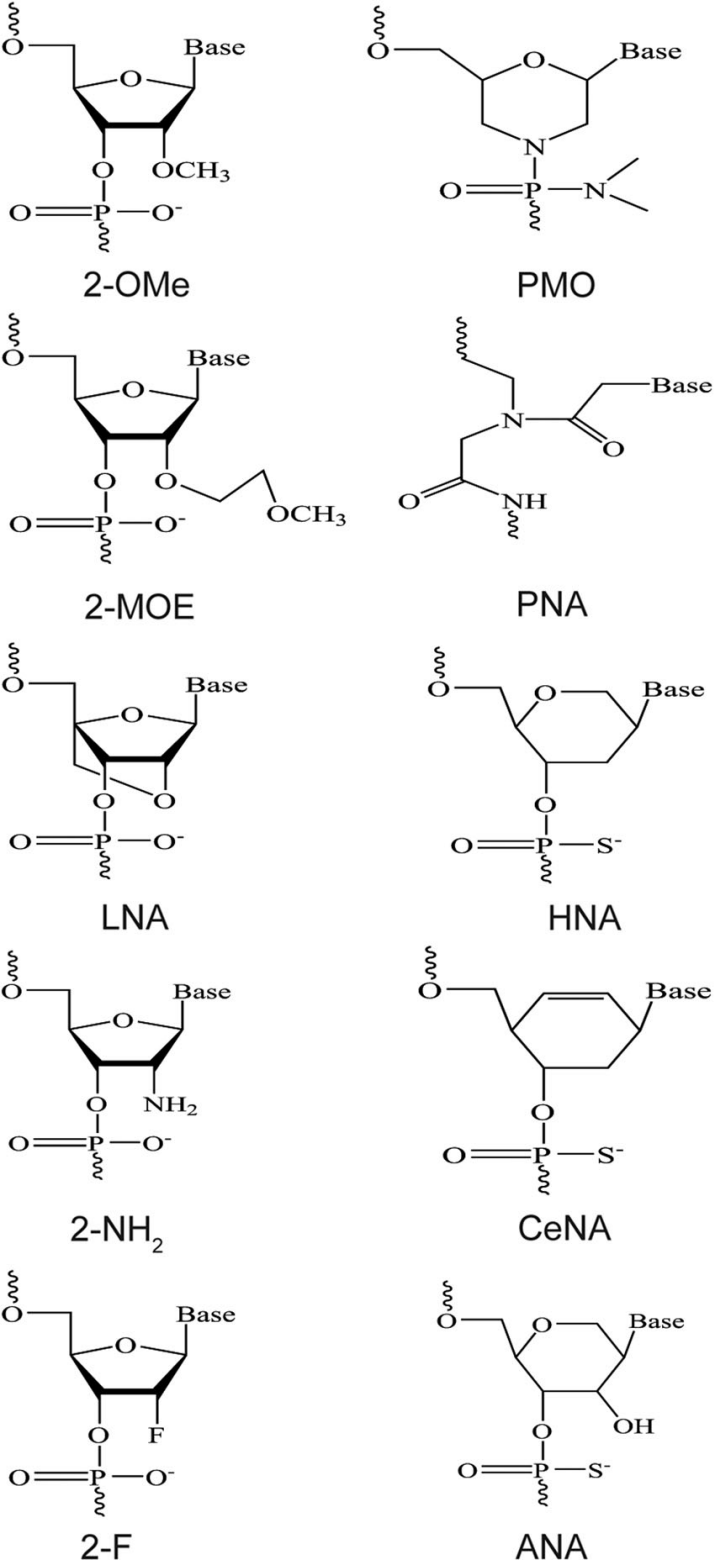
Examples of chemical modifications of nucleic acid analog oligomers. 2‐F, 2′‐fluoro; 2‐MOE, 2′‐*O*‐methoxyethyl; 2‐NH_2_, 2′‐amino; 2‐OMe, 2′‐*O*‐methyl; ANA, altritol nucleic acid; CeNA, cyclohexene nucleic acid; HNA, hexitol nucleic acid; LNA, locked nucleic acid; PMO, phosphorodiamidate morpholino oligomer; PNA, peptide nucleic acid

Chemical modifications (Figure 2) to the backbone and/or bases have been developed to enhance nuclease resistance and overall pharmacokinetic characteristics. The replacement of the nonbridging oxygen atom in the phosphodiester backbone with a sulfur atom creates a phosphorothioate linkage with enhanced resistance to degradation.^135^, ^136^ Furthermore, modifications at the 2′‐position include replacing the hydroxyl group with an *O*‐methyl (OMe), *O*‐methoxyethyl (MOE), fluoro (F), or amino (NH_2_) moiety, or linking the 2′ oxygen to the 4′ carbon to create locked nucleic acid (LNA). These chemical modifications not only increase the nuclease resistance but also the binding affinity of ASOs, siRNAs, aptamers, or other nucleic acids.^137^, ^138^, ^139^, ^140^ Modifications on both sugar and backbone such as peptide nucleic acid (PNA)^141^ and phosphorodiamidate morpholino oligonucleotide (PMO)^142^ have also been developed to enhance oligomer stability. Building on a backbone of morpholine rings connected by phosphorodiamidate linkages, the neutrally charged PMO has been shown to be a very safe chemistry in clinical trials, although a delivery system is needed to allow for better uptake of PMO.^143^ Newer modifications including altritol nucleic acid, twisted intercalating nucleic acid, cyclohexeny nucleic acid, and hexitol nucleic acid are shown to have higher exon skipping efficiency compared with 2‐OMe ASO in vitro,^144^, ^145^ however, the toxicities and in vivo safety profile of these chemistries remain undetermined.

Actually, most chemical modifications incorporated into oligonucleotides result in drastic toxic consequences.^146^ The phosphorothioate backbone has been demonstrated to confer innate immunostimulatory activity and activate the complement system.^147^, ^148^, ^149^ Significant upregulation of immune system associated genes was observed in mouse brains after intracerebroventricular (ICV) administration of 2‐OMe ASO on the phosphorothioate backbone.^150^ Chronic intravenous infusion of 2‐MOE modified oligomers on the phosphorothioate backbone in cynomolgus monkeys results in increased plasma concentration of the complement split product inducing Bb, C3a, and C5a, indicating activation of the alternative pathway of the complement system.^149^, ^151^ However, oligonucleotide‐induced complement activation was not observed in dog and human.^152^

Due to their polyanionic nature, phosphorothioate backbone oligomers are known to bind to many serum proteins, including intracellular and extracellular receptors, causing renal or hepatic toxicities. For example, LNA‐related hepatoxicity is speculated to be caused by LNAs aptameric binding to hepatic intracellular proteins.^153^, ^154^ Specific oligonucleotide sequence motifs, such as TGC or TCC motifs of LNAs in a 3–8–3 gapmer design, tend to exhibit a high propensity to bind to mouse liver proteins and increase hepatotoxicity.^154^ Binding with proteins in proximal tubule cells is also suspected of contributing to the LNA‐associated nephrotoxicity. However, the accumulation of the LNAs within proximal tubule lysosomes is more likely to cause renal toxicity,^155^ as shown by tubular necrosis and oligomer accumulation in the kidney biopsy from the first human trial of an LNA ASO.^156^

### 3.2 Off‐target effects of nucleic acid oligomers

Nucleic acid oligomers bind to targeted sequences in a highly specific base pairing manner, however, off‐target annealing to an unintended RNA that shows some homology is still one of the concerns over the safety of the oligonucleotide compounds. siRNAs inevitably cause unintended gene silencing due to miRNA‐like effects, because both exogenous siRNAs, including short hairpin RNAs (shRNAs) that eventually produce siRNAs, and endogenous miRNAs share the same downstream effector.^157^ After loading with the effector and the Ago protein and then partial pairing to the 3′‐untranslated region (UTR), siRNAs intrinsically may suppress up to 1,000 unintended gene transcripts.^158^ Such off‐target effects show the same dose‐response as on‐target effects, thus it is suggested that siRNA treatment should be titrated down to levels that maintain sufficient on‐target effects.^159^ However, simply reducing concentration is not sufficient to eliminate off‐target effects. Since the hydroxyl group at siRNA nucleotide position 2 is essential to form a hydrogen bond with the asparagine residue of Ago,^160^ chemical modifications on that 2′‐position could render steric constraints and interfere siRNA–Ago interaction, thus reducing siRNA off‐target effects. Inducing 2‐OMe at siRNA positions 1 and 2 is found to reduce around 80% of putative off‐target transcripts, resulting in a 66% decrease in unintended gene silencing.^157^ Similar reduction in off‐target effects is observed when siRNA is chemically modified as LNA or unlocked nucleic acid,^161^ however, the safety profile of these chemistries may hinder their further application. Novel strategies such as making circular siRNAs^162^ or adding a biotin group at the 5′‐end of the sense strand^163^ have been shown to improve siRNA specificity and reduce off‐target effects.

Off‐target effects of RNase H‐inducing ASO have also been known for decades, and increased oligonucleotide binding affinity by chemical modifications may potentially exaggerate the unwanted effects.^164^ Reducing the overall binding affinity by limiting the number of LNAs in a gapmer ASO or increasing the length of ASO to make more specific binding to the target sequence have been shown as potential strategies to mitigate off‐target binding.^165^ Splice‐switching ASOs were believed to have less off‐target effects because they must stringently bind to short splicing motifs such as exonic splicing enhancers or intronic splicing silencers. Moving one or a few nucleotides upstream or downstream of these splicing motifs may result in no splice‐switching activity.^166^, ^167^ However, in a recent study 17 missplicing events were detected by reverse transcription‐polymerase chain reaction after in vitro transfection of one uniformly modified 2‐MOE splice‐switching ASO, with that ASO predicted to have over 108 potential annealing sites in addition to its intended target.^168^ Strategically placing mismatches within the ASO or combining two short ASOs were shown to reduce off‐target effects to some extent. Furthermore, delivering ASO through free uptake markedly reduced off‐target effects compared with delivering through lipid transfection, which may indicate that splice‐switching ASO off‐target effects are a greater concern in vitro than in vivo.^168^ Since there are only a few reports on splice‐switching ASO induced missplicing activity, additional investigations are needed to assess global off‐target effects. Developing reliable in silico tools to predict off‐target effects^169^, ^170^ would allow for identifying and avoiding unwanted oligonucleotide actions before in vitro and in vivo evaluation.

As with any drug, effective delivery is of paramount importance to its therapeutic potential. Potent nucleic acid delivery systems are also needed to ensure effective cell uptake or even tissue‐specific delivery to allow for better assessment of therapeutic benefits. Various approaches, especially bioconjugates including cholesterol, cell‐penetrating peptides, nucleolipids, receptor ligands, and antibodies have been proposed to enhance nucleic acids delivery,^171^, ^172^, ^173^, ^174^ however even those approaches have limitations including immunogenic consequences and lack of efficiency and specificity.^175^ To date, *N*‐acetylgalactosamine (GalNac) appears to be the most potent conjugate for siRNA delivery that specifically targets hepatocytes with limited off‐target effects.^176^, ^177^ Targeted ASO delivery was also achieved by conjugating ASO with GalNAc, resulting in selective ASO uptake by hepatocellular carcinoma cells. Systemically administered GalNAc‐conjugated ASO demonstrated enhanced antisense mediated antitumor activity both in vitro and in vivo.^178^ However, up to now, cell‐penetrating peptides emerge as the most suitable vehicle for ASO systemic delivery which leads to two cell‐penetrating peptides conjugated splice‐switching ASOs, Eteplirsen^179^ and Golodirsen^180^ being approved by the FDA.

The development of nucleic acid therapeutics is rapidly evolving with many issues regarding drug stability, toxicity, off‐target effects, drug delivery being resolved to some extent, dramatically bringing these therapies closer to patients. The recent FDA approvals of *Exondys 51* ^®^, *Spinraza* ^®^, *Tegsedi* ^®^, *Onpattro* ^®^, *Vyondys 53* ^®^, *Milasen* ^®^, and *Givlaari* ^®^ illustrate the broad therapeutic potential of nucleic acid drugs for the treatment of neurological and neuromuscular diseases. Consequently, an increasing number of researchers are actively investigating the therapeutic effects of different nucleic acid compounds for neurodegenerative diseases, including PD. Table 2 shows the current status of potential nucleic acid therapeutic strategies for PD precision or personalized medicines.

### 3.3 Progress in nucleic acid therapeutics for PD

#### 3.3.1 Antisense oligonucleotides

**Figure 3:**
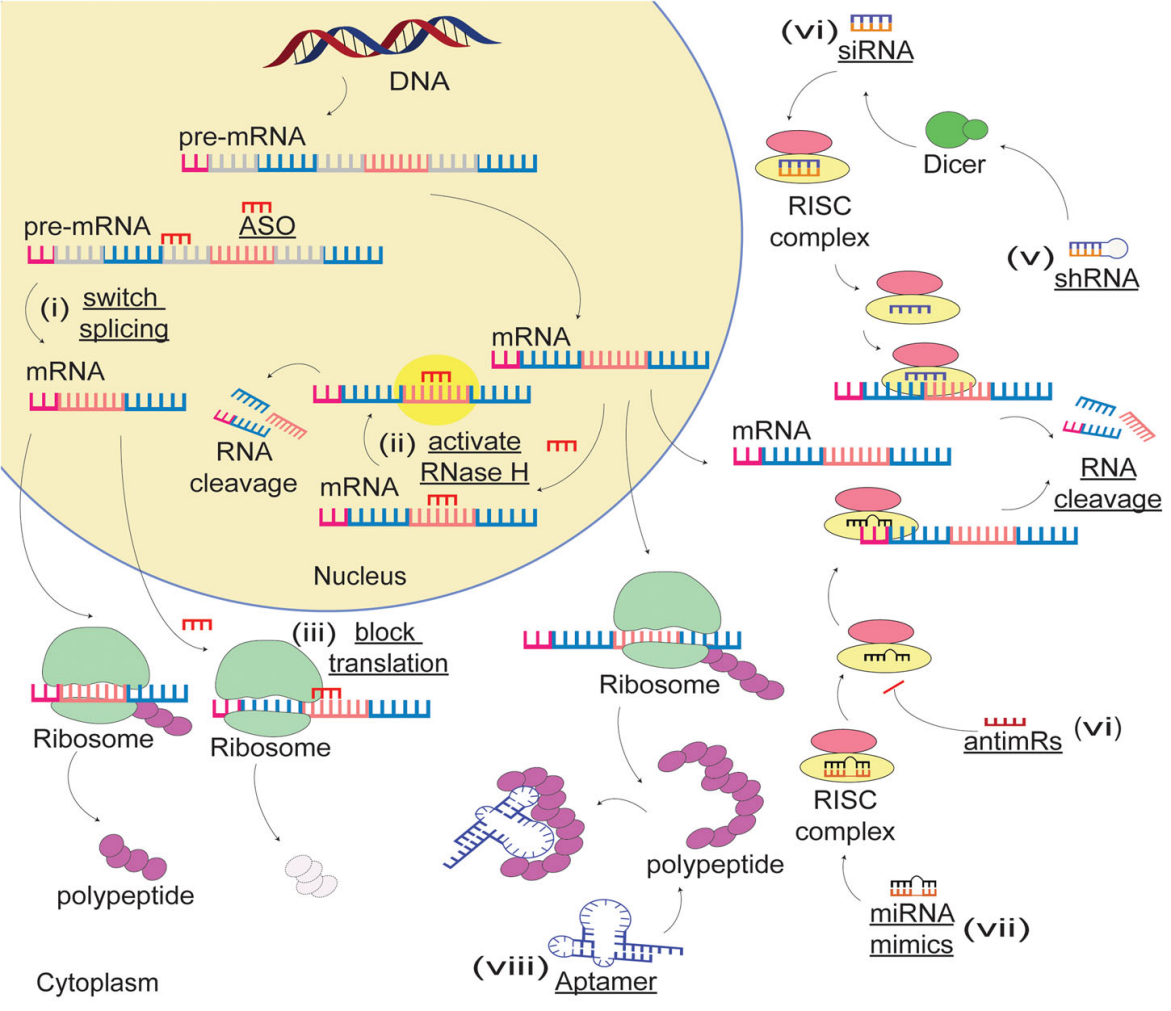
Examples of the mechanism of actions of nucleic acid compounds. (i) ASO‐mediated splice‐switching; ASOs bind to the acceptor or donor splice site, switch splicing, and induce alternative mRNA and protein isoforms; (ii) ASOs activate RNase H and cleave mRNA; (iii) ASOs inhibit mRNA translation by steric blockade of ribosomes; (iv) siRNA‐induced gene silencing; after being taken up by the RNA‐induced silencing complex and ejection of the passenger strand, the antisense strand of siRNA binds to mRNA and mediates mRNA cleavage; (v) shRNA‐mediated targeted gene silencing; (vi) miRNA‐induced RNA cleavage; (vii and viii) mechanism of actions of antimRs and aptamers. ASO, antisense oligonucleotides; miRNA, microRNA; mRNA, messenger RNA; RISC, RNA‐induced silencing complex; shRNA, short hairpin RNA; siRNA, small interfering RNA [Color figure can be viewed at wileyonlinelibrary.com]

ASOs are synthetic nucleic acid analogs that are designed to anneal to target RNA transcripts through Watson–Crick base pairing. Depending upon the base and backbone chemistries, ASOs can modify gene expression through inducing a variety of mechanisms including suppressing translation, altering splicing, modifying polyadenylation, or degrading RNA by activating RNase H or inducing RNA silencing (Figure 3i–iii). In situations where a gene lesion results in a misfolded toxic protein and the abnormal protein contributes to the disease pathway, RNA silencing or RNase H activating ASOs can be designed to bind to and degrade the corresponding messenger RNA (mRNA). The reduction in the targeted mRNA would subsequently reduce the protein levels and slow the disease course, as demonstrated by the development of *Tegsedi* ^®^ for patients with hereditary transthyretin amyloidosis (HTA).^181^

Given that pharmacological inhibition of LRRK2 could abate α‐synuclein‐induced neurodegeneration,^182^ a 2‐MOE ASO was designed to target *LRRK2* mRNA for RNAse H‐mediated degradation to ameliorate pathologic α‐synuclein inclusion‐body formation. After the administration of the 2‐MOE ASO in a PD mouse model, reduced levels of *LRRK2* mRNA and protein were evident, with diminished pathological aggregation of α‐synuclein in the substantia nigra of treated mice.^183^ Since August 2019, a Phase I clinical trial (NCT03976349) has begun to evaluate this 2‐MOE ASO (BIIB094) through intrathecal administration in PD patients with or without a PD‐related *LRRK2* mutation. This would be the first compound being tested and evaluated in PD patients with a defined genetic cause and some positive results were reported.^184^

An oligonucleotide with amido‐bridged nucleic acid‐modified bases at each end and a DNA core to induce RNase H activity was designed to specifically degrade the human *SNCA* transcript. This compound reduced *SNCA* expression significantly and improved motor functions in a PD transgenic mouse model.^185^ Due to the high prevalence of *GBA* mutations in PD patients, several therapeutic strategies including gene therapy and small molecules are under development for patients carrying this particular PD risk.^186^ Chamishi Therapeutics was founded in September 2019 with a specific focus on developing ASO therapies for GBA‐associated PD.

It is well‐established that alternative splicing events are overrepresented in the brain,^187^ with even subtle DNA variations able to catastrophically disrupt normal pre‐mRNA splicing.^188^ Intense efforts are underway to develop ASO molecules capable of redirecting pre‐mRNA splicing by either promoting retention of a missing exon, excising a disease‐causing exon from the mature mRNA, or removing an exon to change the ratio between different transcripts.^189^, ^190^ α‐Synuclein has four isoforms that are generated by alternative splicing of *SNCA* exons 4, 6, or both.^191^ The isoform lacking exon 6 has higher aggregation propensity than full‐length α‐synuclein, while the isoform lacking exon 4 confers a protective effect.^191^ Thus, changing the ratio between the full‐length α‐synuclein and other isoforms by splice‐switching ASOs may initiate a new pathway for the development of disease‐modifying therapeutics for PD.

The recently reported one‐step strategy to convert astrocytes to functional neurons in situ by RNase H‐inducing ASOs is probably one of the most exciting advances in PD therapeutic research. Professor Xiangdong Fu's group designed ASOs on the phosphorothioate backbone to knockdown polypyrimidine tract‐binding protein 1, which successfully converted primary astrocytes to dopaminergic neurons in the mouse brain.^192^ The converted dopaminergic neurons were shown to progressively mature and replenish lost dopaminergic neurons in a PD mouse model, thereby restoring striatal dopamine and reversing motor phenotypes.^192^ This study could open up a completely novel avenue for development of treatments to “rebuild” damaged brains in PD, however, there are still some uncertainties about the effectiveness of this treatment in older individuals with reduced brain plasticity because of age.^193^

#### 3.3.2 Small interfering RNA

Twenty years after the first description of RNA interference (RNAi), the first RNAi drug *Onpattro* ^®^ was approved by the FDA for the treatment of HTA in 2018.^194^, ^195^ The following year, the FDA approved the second siRNA drug *Givlaari* ^®^ to treat patients with acute hepatic porphyria.^196^ RNAi is a process that inhibits targeted gene expression with high specificity and is an exciting therapeutic strategy for precision medicine. siRNAs are generally synthetic, chemically modified short double‐stranded RNAs that are usually 21–23 nucleotides in length. siRNAs contain an antisense active strand and a sense mRNA sequence that is ejected when the double‐stranded siRNAs are taken up by the endogenous cytoplasmic RNA‐induced silencing complex. The active strand can bind to the target mRNA and initiate sequence‐specific mRNA degradation by the RNA‐induced silencing complex argonaute RNase (Figure 3iv). As with ASOs, chemical modifications of the 2′‐ribose and the phosphorothioate backbone help protect siRNAs from nuclease degradation and prolong its half‐life.^139^ Incorporating siRNA into different lipid nanoparticles, especially second‐generation lipid nanoparticles, for example, *Onpattro* ^®^, substantially improves siRNA delivery and gene knockdown.^197^, ^198^ Other conjugates such as GalNac, lipoproteins, or exosomes are also showing improved siRNA delivery and increased knockdown of targeted genes.

*SNCA* overexpression and accumulation of α‐synuclein protein plays a crucial role in the pathogenesis of PD, particularly in families with *SNCA* copy number variations.^199^, ^200^ Thus, reducing *SNCA* mRNA levels and the resultant protein by siRNA is considered as a viable therapeutic strategy for PD. Rabies virus glycoprotein (RVG) decorated anionic liposomes loaded with siRNA‐protamine complex were used to silence *SNCA* expression. This complex was rapidly taken up by primary neuronal cells and efficiently reduced α‐synuclein protein in mouse primary cortical and hippocampal neurons.^201^ In other preclinical studies, polyethylenimine‐siRNA and RVG‐exosome‐siRNA targeting α‐synuclein transcripts also demonstrated a robust reduction of both overexpressed human *SNCA* mRNA and α‐synuclein protein in the striatum of a PD mouse model and delayed the development of α‐synuclein pathology.^202^, ^203^ However, a delicate *SNCA* level must be maintained as it is obvious that too little α‐synuclein may also be harmful to the brain because of its essential biological functions at the synapse. Marked downregulation of *SNCA* expression by siRNA was associated with an increased risk of developing nigrostriatal degeneration.^204^ Therefore, expression‐control siRNAs were generated by introducing nucleotide mismatches to control the levels of *SNCA* knockdown. Administration of various expression‐control siRNAs in PD transgenic fly models showed different levels of *SNCA* knockdown that correlated with motor function improvement.^205^

#### 3.3.3 Short hairpin RNAs

shRNAs are RNA transcripts that contain a loop structure and a stem that consists of a paired sense and antisense strand. As distinct from siRNAs, bacterial or viral vectors are needed to deliver shRNAs into cells, where they are processed into siRNAs and mediate targeted gene inhibition by the RNAi machinery (Figure 3v). Since host cells can continuously synthesize shRNAs, they have several advantages over siRNA including longer‐lasting effects, lower dose requirement, and less specific and nonspecific off‐target effects.^206^ However, there are also some disadvantages to use viral vectors, including immunogenicity and potential risk of inducing mutations, making viral vectors potential safety hazards.^207^ Adeno‐associated virus‐mediated delivery of shRNAs in normal rats resulted in a 35% knockdown of α‐synuclein without affecting motor function or causing degeneration of dopaminergic neurons.^208^ The α‐synuclein knockdown by shRNAs in a PD rat model was observed to be neuroprotective, decreasing the degeneration of dopaminergic neurons and attenuating the progression of motor deficits.^208^ shRNAs have also been used to knockdown polypyrimidine tract‐binding protein 1 and convert astrocytes to dopaminergic neurons in a PD mouse model, thereby replacing dopaminergic neuron loss and reversing motor deficits.^192^ This approach has the potential to open up new therapeutic avenues for PD and other neurodegenerative disorders, but more in vivo and preclinical investigations will be required.

Since most viral vectors do not cross the blood–brain barrier, nanoparticles are being evaluated for delivery of shRNAs to the brain. *N*‐isopropylacrylamide is one type of nanopolymer material capable of controlled cargo targeting and release and is usually combined with acrylic acid as a drug delivery vehicle. This vehicle was further combined with photoactive nerve growth factor and oleic‐coated magnetic nanoparticles to deliver shRNAs targeting *SNCA* across the blood–brain barrier. Subsequently, the shRNAs reduced α‐synuclein expression and prevented dopamine neuron degeneration in the 1‐methyl‐4‐phenyl‐1,2,3,6‐tetrahydropyridine PD mouse model.^209^ Instead of directly targeting *SNCA*, other approaches, including reducing inflammation by shRNA‐mediated silencing of caspase‐1^210^ or class II transactivator have also been attempted.^211^ In addition, inhibition of *Nurr1* ^212^ or *Shp‐2* ^213^ by shRNAs was designed as a potential strategy for the management of levodopa‐induced dyskinesias. However, since Nurr1 has multiple functions including protection of dopaminergic neurons against neurotoxins and suppressing neuroinflammation,^214^ there is still much research needed before the Nurr1‐based PD therapeutics can be considered for the treatment of PD.

#### 3.3.4 miRNA therapeutics

miRNAs are short noncoding RNAs, generally, 20–25 nucleotides in length, that regulate gene expression through binding to the 3′‐UTR of a targeted mRNA. Understanding the critical roles of miRNAs in cellular and molecular biology,^215^ and unveiling the mechanisms of dysregulation of miRNAs under disease conditions^216^, ^217^, ^218^ has made these molecules very attractive propositions with respect to drug development. Since the first discovery of mammalian miRNAs in 2000, rapid progress in RNA chemistry and delivery technologies has enabled multiple miRNA‐based therapeutic compounds to move into clinical trials.^140^ miRNA molecules can be synthesized with distinct chemical modifications that determine the mode of actions (Figure 3vi,vii). miR mimics are double‐stranded short RNA molecules and are often modified by *O‐*methylation of the passenger strand to increase stability.^140^ When delivered to the targeted tissue, miR mimics can replenish the expression of downregulated miRNAs and silence targeted mRNA expression.^216^ On the other hand, single‐stranded antimiRs (also called antagomiRs) either incorporating LNA or 2‐OME residues can inhibit specific miRNAs and upregulate targeted mRNA expression.^219^ In conjunction with antimiRs, miRNA “sponges” or “decoys” are other strategies that can inhibit miRNA activity by providing multiple target sites complementary to specific miRNAs, and subsequently prevent miRNAs binding to endogenous target genes.^220^, ^221^

Studies have shown that miR‐7 regulates neuroinflammation and can also bind directly to specific regions of the α‐synuclein 3′‐UTR and repress α‐synuclein expression.^222^, ^223^ Injecting miR‐7 mimics directly into mouse striatum suppressed inflammasome activation and attenuated dopaminergic cell death.^224^ Using a miR‐decoy stably reduced miR‐7 function and resulted in an increased expression of α‐synuclein and a rapid loss of dopaminergic neurons in vivo.^225^ Other miRNAs that could downregulate α‐synuclein expression include miR‐153 and miR‐214, the latter also participating in brain repair in PD mouse models.^222^, ^226^, ^227^ miR‐132 regulates embryonic stem cell differentiation into dopaminergic cells, while miR‐132 was upregulated in a PD mouse model and reduced dopamine neuron differentiation. The miRNA sponge mmu‐circRNA‐0003292 is hypothesized to sequester miR‐132 and affect PD pathogenesis,^228^ however, further experimental verification is needed.

The miRNAs usually have a wide range of targets and a single miRNA can exert effects and influence multiple different cellular and molecular pathways. For example, transgenic mice overexpressing miR‐7 showed impaired insulin secretion and a decline in the expression level of transcriptional factors for pancreatic islet β‐cell differentiation and developed diabetes.^229^ Over 1,000 genes were found to be dysregulated in an unbiased genome‐wide expression assay in a pancreatic β‐cell line after adenovirus delivered miR‐7 treatment.^229^ Thus, before bringing miRNA therapeutics into clinical applications, potential perturbations to other body systems must be thoroughly investigated. In addition, unlike the application of miRNAs therapeutics in liver disease, where miRNA mimics or antimiRs may readily enter the target tissue and exert their effects,^230^ difficulties in miRNA delivery across the blood–brain barrier is one of the factors that limit the progress in miRNA‐based therapeutics^231^ and other therapeutic nucleic acids. Different miRNA profiles are found in PD and other neurodegenerative disorders, with increased expression of miR‐30a‐5p, miR‐153, and miR‐4639‐5p being identified in PD compared with unaffected healthy individuals.^232^, ^233^, ^234^ Thus, it is likely that, in the near future, the application of miRNA panels on body‐fluids such as a serum, saliva, and cerebrospinal fluid may provide potential PD diagnostic biomarkers, and may also help stratify PD patients according to the genetic and molecular pathways involved.

#### 3.3.5 Aptamers

Aptamers are single‐stranded DNA or RNA molecules that fold into defined three‐dimensional (3D) structures for specific target binding (Figure 3viii). Aptamers are generally selected from large DNA or RNA oligonucleotide libraries by systematic evolution of ligands by exponential enrichment.^235^ The specific 3D configuration of these small molecules enables selected aptamers to bind to specific targets in a lock‐key manner.^236^ Thus, aptamers have a wide range of potential applications including diagnostics, biomarker discovery, and targeted therapeutics where aptamers can be divided into three groups: antagonist, agonist, and carrier for other therapeutic compounds. Including the first FDA approved RNA aptamer drug *Macugen* ^®^, all aptamers currently in clinical trials were designed to function as antagonists.

The first DNA aptamer selected to bind to α‐synuclein initiated the exploration of these compounds in PD research, including investigating the pathogenesis and developing diagnostics and therapeutics for PD.^237^ Since α‐synuclein oligomers are reported to be cytotoxic and are likely to play a major role in PD pathogenesis,^238^ DNA aptamers were selected to bind to α‐synuclein oligomers specifically, rather than monomers or fibrils.^239^ In light of additional evidence that antibodies targeting the C‐terminus of α‐synuclein reduced the extent of oligomerization and ameliorated nigral dopaminergic neuron loss,^240^, ^241^ two aptamers selected from 11, 019 sequences were found to bind to α‐synuclein with high affinity.^242^ After entry into SK‐N‐SH cells and primary neurons, these peptide‐conjugated aptamers inhibited α‐synuclein aggregation, promoted α‐synuclein clearance, protected against α‐synuclein‐induced mitochondrial dysfunction, and rescued the cell defects.^242^

Aptamers have also been applied to monitor dopamine concentration, with “Apta‐sensors” including the ultrasensitive and selective voltammetric apta‐sensor,^243^ and gold nanoparticle enhanced surface plasmon resonance apta‐sensor being reported to quantitate dopamine levels down to 200 fM.^244^ This superb level of sensitivity and specificity in detecting dopamine supports the development of apta‐sensors as tools for clinical diagnostics and monitoring disease progression in clinical trials. Although aptamer therapies have some disadvantages, including rapid clearance, metabolic instability, and poor translation in in vivo studies,^245^, ^246^ aptamers are believed to have considerable potential as novel therapeutics with advanced biological functions, once aptamer designs and modifications are optimized.

## 4 CHALLENGES IN NUCLEIC ACID THERAPEUTICS FOR PD

**Figure 4:**
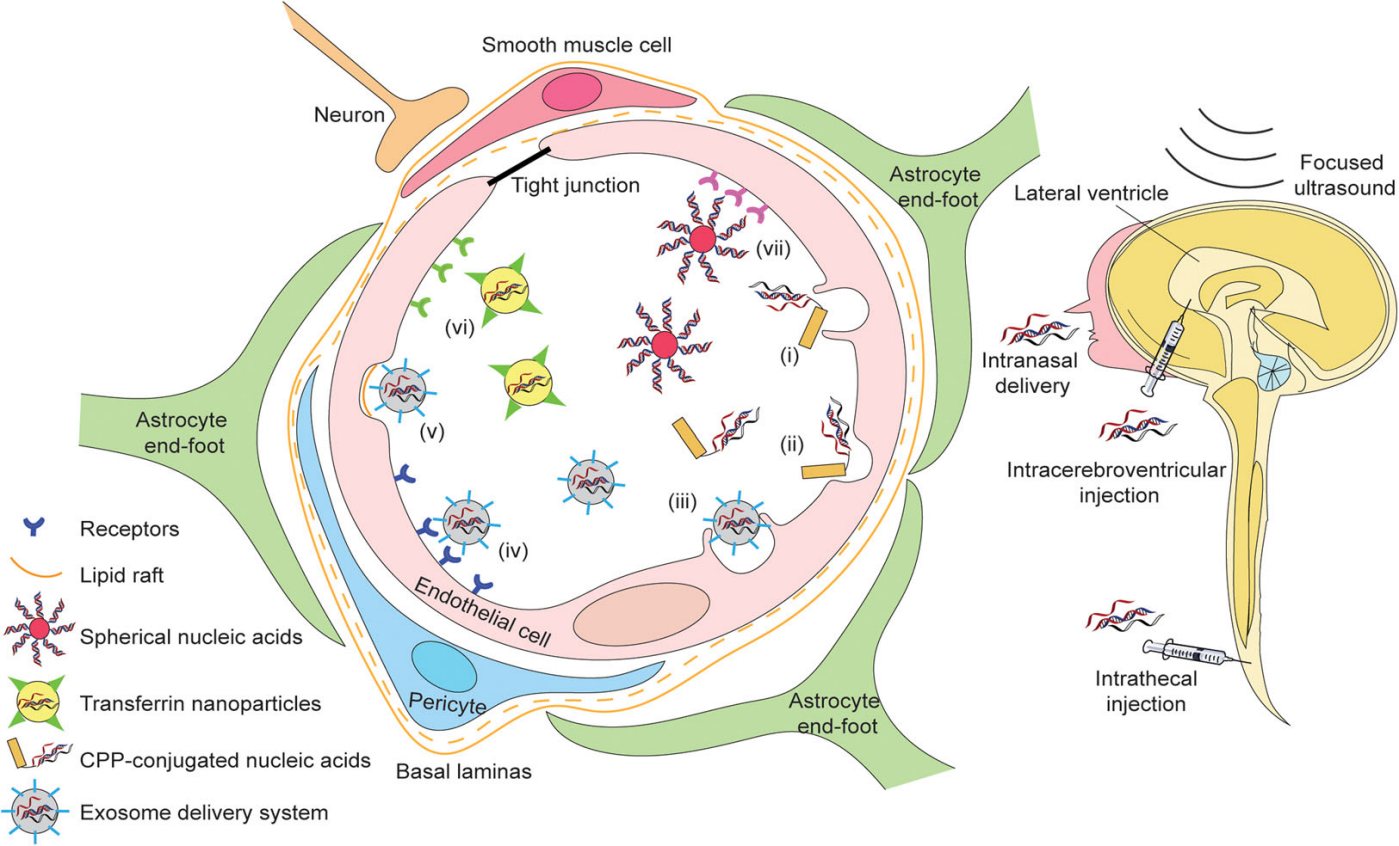
Strategies for the delivery of nucleic acid drugs across the blood–brain barrier into the central nervous system. The blood–brain barrier is formed by the cerebral endothelial cells with tight junctions at their margins, pericytes, basal lamina, and astrocytic end‐feet. Intracerebroventricular and intrathecal injection or infusion can directly administer compounds, including nucleic acids, into the central nervous system. Through intranasal administration, nucleic acid drugs can enter the central nervous system through the nose‐to‐brain route, mainly mediated by the olfactory and trigeminal nerve pathways.^250^ Cell‐penetrating peptide‐conjugated nucleic acids are taken up through macropinocytosis (i) and endocytosis (ii) by the endothelial cells. The exosome system delivers nucleic acids across the blood–brain barrier mainly through receptor‐mediated endocytosis (iv), lipid‐raft mediated endocytosis (v), and macropinocytosis (iii). Receptor‐mediated endocytosis also contributes to the uptake of transferrin nanoparticles (vi) and spherical nucleic acids (vii) [Color figure can be viewed at wileyonlinelibrary.com]

The first and foremost obstacle for the application of nucleic acid therapeutics for PD and other CNS disorders is the blood–brain barrier. Blood‐to‐brain transporters have been shown to facilitate transport of systemically‐delivered ASOs across the blood–brain barrier, and consequently bring a certain level of therapeutic benefit in animal models of Alzheimer's disease^247^ and stroke.^248^ However, the amount of ASOs that reached the brain in these studies was less than 1% of the dose administered intravenously.^249^ Several strategies have been investigated to address the blood–brain barrier and increase drug delivery into the CNS, including direct CNS administration and the vehicle cargo drug delivery system, as illustrated in Figure 4.

ICV injection/infusion is considered to be a safe and long‐term route for CNS drug administration.^251^ ASOs composed of 2‐OMe modified bases on a phosphorothioate backbone were shown to be evenly distributed in the parenchyma surrounding the ventricles in the mouse brain within a few hours after ICV infusion.^252^ These ASOs were taken up preferentially by neurons in key brain structures such as the cerebellum, striatum, hippocampus, and dentate gyrus.^252^ Although the pharmacokinetics and distribution pattern of other chemical modifications might be slightly different, ICV‐delivered ASOs using several different chemistries are generally showing therapeutic potential in preclinical studies. Continuous ICV infusion of a 2‐MOE phosphorothioate ASO was reported to mediate a sustained reversal of the Huntington's disease phenotype.^253^ Furthermore, persisting allele‐specific mutant *HTT* gene silencing was achieved by ICV injection of constrained ethyl (cEt) ASOs.^254^ However, the chance of adverse events, including infection and tissue damage induced by ICV cannot be ignored. The possibility of activation of the innate immune system, as was detected after ICV administration of 2‐OMe ASOs in mice,^255^ is another factor that may eventually restrict the long term, repeated ICV ASO delivery in clinical studies.

Since the clinical trial (NCT01041222) using intrathecal delivery of an ASO to treat familial amyotrophic lateral sclerosis caused by SOD1 mutations,^256^ most of the clinical trials for CNS indications are using intrathecal delivery.^130^ Intrathecal injection of Nusinersen to infants and children with spinal muscular dystrophy was shown to be safe and tolerable.^257^ However, as ICV and intrathecal drug delivery are both relatively invasive procedures, intranasal administration is an alternative approach that could deliver nucleic acid compounds to the CNS through endocytosis across the permeable olfactory epithelium.^250^, ^258^ Intranasally administered sertraline‐conjugated siRNA was internalized into 5‐hydroxytryptamine neurons and reduced serotonin transporter expression in raphe nuclei, evoking fast antidepressant‐like responses in mice.^258^ In addition, intranasal delivery of LNA antagomirs targeting miR‐210 decreased brain miR‐210 levels and improved neurological function later in life, which outcomes were similar to those resulting from ICV‐mediated ASO treatment in a neonatal rat model.^259^

In addition to selecting optimal delivery routes to achieve expected CNS therapeutic effects, multiple novel vehicle cargo drug delivery systems are being developed. One promising delivery approach is to conjugate cell‐penetrating peptides that penetrate the blood–brain barrier to the ASOs, thereby facilitating the uptake and actions of nucleic acid compounds. A feature of many of these peptides is the presence of tracts of arginine residues interrupted by short hydrophobic sequences.^260^ PNA/PMO internalization peptide 6a (Pip6a), derived from the Penetratin peptide, consists of a central hydrophobic core flanked on either side by arginine‐rich sequences.^261^ Pip6a‐PMO was shown to not only be taken up by muscle cells through endocytosis in vitro,^262^ but also increased *SMN* expression in CNS tissues and resulted in profound phenotypic correction in SMA mice.^263^ However, toxic effects, especially nephrotoxicity, remains as the main challenge to this category of peptide reaching clinical application.^264^

Exosomes are endocytic nano‐vesicles that could enter the CNS through transcytosis or internalization by endothelial cells^265^ and thus have been designed as drug vehicles.^266^ Several preclinical studies are using exosomes to deliver siRNAs for neurological diseases, including Huntington's disease^267^ and glioblastoma multiforme with few toxic effects or immunogenicity observed after repeated doses.^268^, ^269^ In one recent study, RVG peptide was fused to lysosome‐associated membrane glycoprotein (Lamp2b) on exosomes, and the RVG‐Lamp2b modified exosome was loaded with miR‐124 mimics. Systemic administration of the RVG‐Lamp2b‐miR‐124 localized to the infarct region in a focal cerebral ischemia model and ameliorated the ischemic injury by promoting neurogenesis.^269^ Exosomes as the carriers for nucleic acid compounds are biocompatible, immunologically inert, and can efficiently reach the target.^270^ However, efficiently and reliably isolating and characterizing exosomes is one of the obstacles that need to be addressed for future clinical applications.

Expanding research on the blood–brain barrier has changed the view on the blood–brain interface and transformed strategies to overcome its impermeability.^271^ Blood–brain barrier‐penetrating peptides bearing the anti‐transferrin receptor antibody^272^ and transferrin‐containing nanoparticles^273^ promote the entry of therapeutic cargos into the CNS by absorptive transcytosis. Other nanoparticle‐nucleic acid delivery systems, such as spherical nucleic acids (gold nanoparticles)^274^ and ASO‐liquid nanocapsules^275^ are also being investigated to deliver nucleic acids across the blood–brain barrier (Figure 4). Spherical nucleic acids are generally composed of gold nanoparticles at the core and a dense layer of thiol‐modified oligonucleotide in the shell, which render spherical nucleic acids more resistant to degradation compared to linear nucleic acids.^276^ More importantly, spherical nucleic acids can be rapidly internalized by any cell type and readily cross the blood–brain barrier when administered systemically.^277^ This nanoparticle system has been given approval by the FDA for delivery of NU‐0129, a spherical nucleic acid gold nanoparticle containing siRNAs targeting the Bcl‐2‐like protein, for an early‐stage clinical trial in glioblastoma multiforme. In its Phase 0 first‐in‐human trial (NCT03020017), initial evidence of crossing blood–brain barrier, with no unexpected adverse effects, further validated this approach for CNS drug delivery.^278^

These studies demonstrate the potential of vehicle cargo delivery systems to enhance the delivery of nucleic acid oligomers across the blood–brain barrier to their targets in the CNS. However, many outstanding challenges remain, particularly relating to the stability of the complex compound, possible adverse effects from off‐target distribution of peptides, toxicity, and efficacy of nanoparticles need to be addressed before these complex compounds can progress to clinical trials. Alternatively, physical methods such as MRI‐guided focused ultrasound‐induced blood–brain barrier opening has been approved as a novel means for CNS drug delivery.^279^ Since enhanced delivery of ASOs to the target brain region was achieved by focused ultrasound,^280^ many clinical trials are now applying MRI‐guided focused ultrasound, and showing safe and reversible opening of the blood–brain barrier.^281^

## 5 CONCLUSIONS AND OUTLOOK

This review describes advances in the understanding of the complex molecular pathogenesis of PD, and the proteins and genes that play key roles in familial PD, as well as the more prevalent sporadic form of the disease. It also draws attention to the importance of biomarker‐driven subtyping of PD as a basis for the application of a precision‐medicine approach to the development of novel therapeutic strategies, including nucleic acid‐based therapeutics. Such an approach is an intuitive and logical way of addressing the problem of heterogeneity in PD and of identifying genetic‐molecular mechanisms that may be more specific to certain disease subtypes and could be targets for novel disease‐modifying therapies.

Nucleic acid therapeutics have demonstrated great diversity and potential for the treatment of various neurological disorders and have several important advantages, including high target specificity, stability, and low toxicity and immunogenicity. Other crucial advantages of nucleic acid therapeutics over other small molecule drugs, include the ease and predictable chemical synthesis of these compounds, efficient drug‐discovery process, and similarities in toxicity and pharmacokinetic profiling.^282^ Within 10 months of identification of the genetic cause in a patient with Batten's disease, *Milasen* *^®^*, a splice‐switching oligomer received FDA approval, demonstrating the potential for rapid design and development of nucleic acid therapeutics. Although *Milasen* *^®^* is a patient‐customized drug development case,^14^ it could serve as an exemplar to open up new avenues for clinical trials and regulation of individualized medicines for rare diseases.^283^ The potential of nucleic acid therapeutics in PD is substantial, and studies to date have already shown that they have the ability to modify the expression of key PD proteins such as α‐synuclein and its specific isoforms. Although environmental factors such as pesticides or metals may participate in α‐synuclein aggregation, or the gastrointestinal microbiome could affect PD drug metabolism or clinical phenotypes, nucleic acid‐based therapeutics represent potentially new pathways for disease‐modifying therapies for PD that could be applicable to both familial and sporadic forms of the disease, and have enormous potential, warranting further investigation and development.

## CONFLICT OF INTERESTS

The authors declare that there are no conflict of interests.
